# Effectiveness of the Stand More AT (SMArT) Work intervention: cluster randomised controlled trial

**DOI:** 10.1136/bmj.k3870

**Published:** 2018-10-10

**Authors:** Charlotte L Edwardson, Tom Yates, Stuart J H Biddle, Melanie J Davies, David W Dunstan, Dale W Esliger, Laura J Gray, Benjamin Jackson, Sophie E O’Connell, Ghazala Waheed, Fehmidah Munir

**Affiliations:** 1 Diabetes Research Centre, University of Leicester, Leicester General Hospital, Leicester LE5 4PW, UK; 2 NIHR Leicester Biomedical Research Centre, Leicester General Hospital, Leicester, UK; 3 Institute for Resilient Regions, University of Southern Queensland, Education City, Springfield Central, QLD, Australia; 4 Leicester Diabetes Centre, University Hospitals of Leicester, Leicester General Hospital, Leicester, UK; 5 School of Public Health, The University of Queensland, Brisbane, QLD, Australia; 6 Baker Heart and Diabetes Institute, Melbourne, VIC, Australia; 7 Department of Medicine, Monash University, Melbourne, VIC, Australia; 8 Department of Epidemiology and Preventive Medicine, Monash University, Melbourne, VIC, Australia; 9 School of Exercise and Nutrition Sciences, Deakin University, Burwood, VIC, Australia; 10 School of Sport Science, Exercise and Health, The University of Western Australia, Perth, WA, Australia; 11 Mary MacKillop Institute for Health Research, The Australian Catholic University, Melbourne, VIC, Australia; 12 School of Sport, Exercise and Health Sciences, Loughborough University, Loughborough, UK; 13 Department of Health Sciences, University of Leicester, Leicester, UK

## Abstract

**Objectives:**

To evaluate the impact of a multicomponent intervention (Stand More AT (SMArT) Work) designed to reduce sitting time on short (three months), medium (six months), and longer term (12 months) changes in occupational, daily, and prolonged sitting, standing, and physical activity, and physical, psychological, and work related health.

**Design:**

Cluster two arm randomised controlled trial.

**Setting:**

National Health Service trust, England.

**Participants:**

37 office clusters (146 participants) of desk based workers: 19 clusters (77 participants) were randomised to the intervention and 18 (69 participants) to control.

**Interventions:**

The intervention group received a height adjustable workstation, a brief seminar with supporting leaflet, workstation instructions with sitting and standing targets, feedback on sitting and physical activity at three time points, posters, action planning and goal setting booklet, self monitoring and prompt tool, and coaching sessions (month 1 and every three months thereafter). The control group continued with usual practice.

**Main outcome measures:**

The primary outcome was occupational sitting time (thigh worn accelerometer). Secondary outcomes were objectively measured daily sitting, prolonged sitting (≥30 minutes), and standing time, physical activity, musculoskeletal problems, self reported work related health (job performance, job satisfaction, work engagement, occupational fatigue, sickness presenteeism, and sickness absenteeism), cognitive function, and self reported psychological measures (mood and affective states, quality of life) assessed at 3, 6, and 12 months. Data were analysed using generalised estimating equation models, accounting for clustering.

**Results:**

A significant difference between groups (in favour of the intervention group) was found in occupational sitting time at 12 months (−83.28 min/workday, 95% confidence interval −116.57 to −49.98, P=0.001). Differences between groups (in favour of the intervention group compared with control) were observed for occupational sitting time at three months (−50.62 min/workday, −78.71 to −22.54, P<0.001) and six months (−64.40 min/workday, −97.31 to −31.50, P<0.001) and daily sitting time at six months (−59.32 min/day, −88.40 to −30.25, P<0.001) and 12 months (−82.39 min/day, −114.54 to −50.26, P=0.001). Group differences (in favour of the intervention group compared with control) were found for prolonged sitting time, standing time, job performance, work engagement, occupational fatigue, sickness presenteeism, daily anxiety, and quality of life. No differences were seen for sickness absenteeism.

**Conclusions:**

SMArT Work successfully reduced sitting time over the short, medium, and longer term, and positive changes were observed in work related and psychological health.

**Trial registration:**

Current Controlled Trials ISRCTN10967042.

**Figure:**
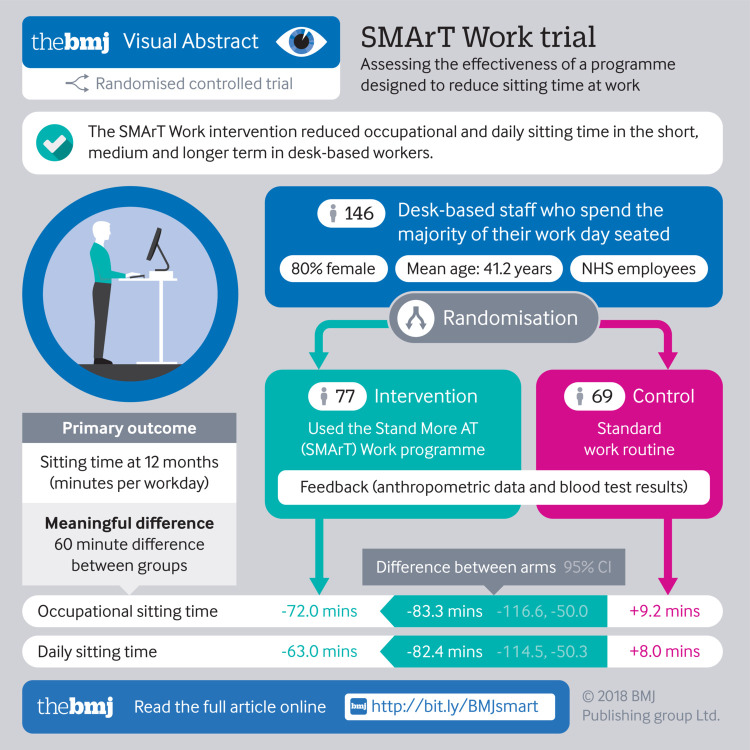


## Introduction

A wealth of epidemiological evidence shows that sedentary behaviour is associated with an increased risk of chronic disease (type 2 diabetes, cardiovascular disease, some cancers) and mortality, often independently of body mass index (BMI) and physical activity,^1^ ^2^ ^3^ ^4^ poor mental health,^5^ ^6^ and a lower quality of life.^7^ Office workers are one of the most sedentary populations, spending 70-85% of time at work sitting.^8^ ^9^ It has also been reported that over a third of their total sitting time at work is accumulated in bouts of prolonged sitting (>30 minutes).^8^ Occupational sedentary behaviour specifically has been associated with an increased risk of diabetes and mortality^10^ and musculoskeletal problems such as neck and shoulder pain,^11^ as well as being detrimental for important work related outcomes such as engagement^12^and presenteeism.^13^ Research on outcomes such as work engagement and presenteeism is, however, limited. These links between sedentary behaviour and health and work related outcomes are important because the estimated costs of presenteeism and absenteeism in the United Kingdom are reported to be more than £30bn ($39bn; €34bn), with presenteeism costing over twice as much as absenteeism.^14^ More positively, reductions in sitting and breaking up sitting through standing and walking in acute experimental settings have led to improvements in important cardiometabolic markers of health such as glucose and insulin levels and blood pressure,^15^ ^16^ ^17^ ^18^ ^19^ ^20^ ^21^ ^22^ and feelings of fatigue and vigour.^23^ ^24^

In response to this evidence, interventions to reduce sitting time in the workplace have received increasing attention in recent years.^25^ These have focused on numerous strategies, including physical changes to the workplace, such as providing height adjustable desks to enable sitting or standing, pedalling workstations, treadmill desks, policy changes, information provision, counselling, and computer prompts.^25^ While positive findings were observed for some strategies in terms of reducing sitting time, particularly the provision of height adjustable desks, the quality of evidence was considered low for most studies owing to non-powered small studies and studies with a high risk of bias.^25^ Furthermore, interventions have typically been evaluated over the short term, so knowledge on longer term effectiveness is lacking. Although some studies have examined the impact of sitting reduction interventions on work related outcomes such as job performance and productivity,^25^ ^26^ presenteeism,^26^ and absenteeism,^26^ ^27^ it is difficult to draw conclusions across these studies owing to the limitations in study designs.

The Stand Up Victoria study was one recent example that addressed these limitations. This was a multicomponent intervention in Australia, and effectiveness was tested within a cluster randomised controlled trial over 12 months.^28^ Components comprised a group based workshop, feedback on sitting behaviour, provision of a height adjustable desk attachment, goal setting, and ongoing support for three months in the form of emails or individual coaching sessions. The intervention was successful in reducing daily sitting and sitting at work^29^ and led to small improvements in glucose levels and cardiometabolic risk.^30^ However, high quality designs remain scarce and studies in the UK are lacking. The Stand More At Work (SMArT Work) intervention was designed in response to this need and was developed using guidance from the Behaviour Change Wheel^31^ (a framework for designing interventions) after formative research with office workers.^32^

We undertook a cluster randomised controlled trial to test the impact of the SMArT Work intervention over the short (three months), medium (six months), and longer term (12 months) in a sample of office workers working within the English National Health Service, the largest employer in the UK. The primary objective was to test whether the SMArT Work intervention led to changes in occupational sitting time at 12 months compared with control.

## Methods

### Study design

The study is reported according to the CONSORT statement for cluster randomised controlled trials. This study was a cluster randomised controlled trial with follow-up measures at 3, 6, and 12 months. The full trial protocol has been published.^33^ Randomisation occurred at the office group level to reduce the risk of contamination. Using computer generated lists, a statistician randomised office groups (clusters) 1:1 to either intervention or control group stratified by cluster size (≤4 and >4 participants) with a block size of six. Randomisation was performed in batches after participant clusters had completed their baseline measures. Team members who took measurements were blinded to group randomisation. The team leads could not be blinded as they were responsible for study coordination, including delivery of the desks and intervention components. Team leads had no involvement in data processing and analysis.

Recruitment took place between October 2015 and June 2016, with baseline data collection between November 2015 and June 2016 and follow-up data collection between March 2016 and June 2017. The study was coordinated from the Leicester Diabetes Centre, University Hospitals of Leicester NHS Trust, and all data were collected on site at the University Hospitals of Leicester NHS Trust.

### Setting and participants

The participants were recruited from the University Hospitals of Leicester NHS Trust. This trust consists of three hospitals across Leicester—Leicester Royal Infirmary, Leicester General Hospital, and Glenfield Hospital. All participants provided informed consent on entering into the study.

During the grant application process, managers across the trust were approached to gauge interest in their team taking part in the study (this information was used to generate the original sample size calculation). Once the study had started, these managers were approached again as well as the staff within their team. Alongside this, we carried out other methods of recruitment. The study was included in the chief executive’s monthly e-newsletter as well as being advertised on the University Hospitals of Leicester NHS Trust staff intranet, and by posters displayed in staff rooms across the hospital sites. To promote the study to staff members and answer any questions they might have, we set up advertisement stands, manned by a member of the research team, in the canteens on each hospital site over lunch times. Any interested teams and individual staff members contacted the study team to obtain a participant information sheet outlining the study requirements and a reply slip used to assess eligibility. Staff members who responded were asked to encourage their colleagues to join the study.

We contacted eligible participants to organise a convenient date to consent them into the study and take their baseline measurements. Measurements were carried out in a private room at the participants’ place of work. Staff aged 18-70 years were eligible if they were office based (self reported and confirmed at a visit by a researcher), spent most (≥75% (self reported), excluding mandatory breaks) of their workday sitting (self reported), worked at least 0.6 full time equivalent, worked at the same desk for at least three days a week, and were capable of standing.

### Participant personal and anthropometric measures

Information on age, sex, ethnicity, smoking status, current job role, pay grade, and working hours were collected by questionnaire. Body weight and body fat (Tanita SC-330ST, Tanita, West Drayton, UK), height (Leicester Height Measure, Seca, Birmingham, UK), and waist circumference (midpoint between the lower costal margin and iliac crest) were measured to the nearest 0.1 kg, 0.1%, 0.5, and 0.5 cm, respectively. Arterial blood pressure was measured in the sitting position (Omron Healthcare, Henfield, UK); three measurements were obtained and the average of the last two used. 

### Outcome measures

Primary and secondary outcomes were assessed at baseline and at 3, 6, and 12 months.

#### Primary outcome

The primary outcome was change in occupational sitting time measured by the activPAL micro (PAL Technologies, Glasgow, UK). The activPAL is a small accelerometer worn on the thigh, which determines body posture—that is, sitting/lying, and upright (with and without stepping). This device is increasingly used in sedentary behaviour research^34^ and has been shown to be highly accurate in measuring sitting, standing, and stepping and in detecting reductions in sitting.^35^ ^36^ ^37^ We asked participants to wear the device continuously for seven consecutive days on the midline anterior aspect of the right thigh. The device was initialised using the manufacturer’s software (activPAL3 Professional Research Edition; PAL Technologies, Glasgow) with default settings. The device was waterproofed with a nitrile sleeve and Hypafix Transparent (BSN medical, Hull, UK) dressing and secured to the thigh with a piece of Hypafix Transparent dressing. We asked participants to complete a log of sleep and wake times while wearing the device, removal times of the device, and the start and end times of each workday.

Devices were collected in person, and a validated algorithm in STATA (StataCorp) was used to download and process data. This has been described elsewhere,^38^ but in brief the algorithm uses the activPAL eventsXYZ.csv files to isolate waking hours from sleeping (time in bed), prolonged periods of non-wear, and invalid data. The processed data were checked visually (by creating heatmaps of the data, as described elsewhere^34^) for any occasions where the algorithm incorrectly coded sleep and waking behaviour (eg, where wake and sleep times were different from other days of data—ie, looked very early or very late compared with other days), and on such occasions we referred to the self reported log and, if necessary, corrected the data. This algorithm has previously shown a high level of agreement with diary reported wear times during waking hours(κ>0.8 for 88% of participants; median κ=0.94).^34^ To isolate data for work hours, we matched the self reported work times collected in the log with those of the device data (in the processed eventsXYZ file). We included events (ie, bouts of sitting, standing, stepping) that crossed the self reported start and end of work times within the work hours data if 50% or more of the event was within the period of interest.^34^ Workplace data were considered valid if the device was worn for 80% or more of self reported work hours^39^ and participants provided at least one valid workday.^29^ To minimise the possibility of reactivity, we discarded the first day of data collected from analysis.

#### Secondary outcomes

*Physical activity and other sedentary behaviour variables*—Other variables of interest calculated from the activPAL data included daily sitting time along with prolonged sitting time (≥30 minutes), standing time, stepping time (light and moderate to vigorous) with outcomes calculated during work hours and daily (ie, across all waking hours). For the daily data we defined a valid day as a day with less than 95% spent in any one behaviour (eg, standing or sitting), more than 500 steps, and 10 hours or more of data from waking hours. To be included in the analysis of daily data we required participants to have at least one valid day.

Alongside the activPAL, participants also wore the ActiGraph Link accelerometer (ActiGraph, Pensacola, FL) on their non-dominant wrist continuously for seven days to capture time spent in moderate to vigorous physical activity during work and daily levels. ActiGraph files were processed with R-package GGIR version 1.5-10 (http://cran.r-project.org).^40^ ^41^ We excluded files from all analyses if post-calibration error was greater than 0.02 *g *(gravity)^42^ or fewer than 10 hours of wear time was recorded during the 24 hour day of interest. Detection of non-wear has been described in detail previously (see ‘‘Procedure for Nonwear Detection’’ in the paper’s supplementary document).^40^ Briefly, non-wear is estimated based on the standard deviation and value range of each axis, calculated for 60 minute windows with 15 minute moving increments. If for at least two of the three axes the standard deviation is less than 13 m*g* (milligravity) or the value range is less than 50 m*g* we classified the time window as non-wear.

We used the threshold of 100 m*g* or more to calculate the time accumulated in moderate to vigorous physical activity at work and daily.^43^

*Musculoskeletal health*—the Standardised Nordic Questionnaire was used to assess musculoskeletal problems in nine body areas (neck, shoulder, upper back, elbow, wrist, lower back, hip, knee, and ankle) over the past week and year.^44^

*Work related measures*—A questionnaire captured several measures. Work engagement was assessed using a nine item questionnaire with a 7-point Likert scale^45^; work engagement is defined by high levels of personal energy where a worker wants to put the time and effort into their work (vigour and vitality) and sees their work as significant (dedication) and interesting (absorption).^45^ Work engagement is an important indicator of both productivity and workforce wellbeing.^46^ Job satisfaction^47^ and performance^48^ were measured using single item questions on a 7-point Likert scale. Occupational fatigue was assessed using the Need for Recovery Scale, an 11 item questionnaire with yes or no options for each question.^49^ The need for recovery refers to the extent that the work task induces a need to recuperate from work induced effort. The severity and duration of symptoms are assessed, which indicate that the respondent is not fully recovered from the effects of sustained effort during the working day and has reduced motivation for activities in the evening with family or friends.^50^ Fatigue at work has been associated with stress and burnout, which in turn can lead to reductions in productivity and higher absence due to sickness.^50^ Sickness presenteeism, often defined as going to work despite illness, was assessed using two questionnaires: the eight item Work Limitations Questionnaire^51^ measured the degree to which health problems interfered with specific aspects of job performance and the productivity impact of these work limitations (presenteeism). It asks employees to rate their level of difficulty (or ability) to perform in eight areas of work in the past two weeks. For example, to concentrate on work, speak with people, handle the workload, and finish on time. Responses are combined into four work limitation scales: time management, physical demands, mental and interpersonal, and output demands. The Work Productivity and Activity Impairment Questionnaire (WPAI-GH 2.0)^52^ measured absenteeism (percentage of work time missed due to health problems in the past seven days), presenteeism (percentage of impairment experienced while at work in the past seven days due to health problems), overall impairment (combination of absenteeism and presenteeism), and activity impairment (percentage of impairment in daily activities as a result of health problems in the past seven days). This latter questionnaire was only used for cost effectiveness analysis and will not be reported in this article. Data on sickness absence from work was obtained by self report (previous three month) and by organisational records for 12 months before the start of the study and for the 12 months’ duration of the study.

*Cognitive function*—Cognitive function was assessed using computerised and paper based tasks. A touch screen laptop was used for the Digit Symbol Substitution Test,^53^ which assesses processing speed, attention, and concentration, and the Stroop Colour-Word Test, which assesses executive function.^54^ Paper based tasks included the Hopkins Verbal Learning Test to assess memory recall^55^ and verbal fluency.^56^

*Mood and affective states*—Mood and affective states were assessed using the Mood Affect Adjective Check List-Revised. This check list measures anxiety, depression, hostility, and positive and sensation seeking affects.^57^ It measures affect both as a temporary state (today) and, more generally, as a disposition (generally).

*Quality of life*—The World Health Organization Quality of Life-BREF was used to measure quality of life. This questionnaire incudes four domains: physical health, psychological health, social relationships, and environment.^58^

### Intervention group

The intervention group received the SMArT Work intervention for the length of the randomised controlled trial (12 months). SMArT Work is grounded in several behaviour change theories (social cognitive theory,^59^ organisational development theory,^60^ habit theory,^61^ self regulation theory,^62^ and relapse prevention theory^63^), and it is implemented through the Behaviour Change Wheel and the associated capability, opportunity, motivation, and behaviour (COM-B) approach.^31^ The intervention design and behaviour change strategies take into account the organisational environment and social norms, individual, and interpersonal factors that influence sitting behaviour at work. Supplementary table 1 provides the timeline of these strategies and supplementary figure 1A includes the logic model of the intervention.

*Organisational strategies*—We sought management buy-in by meeting with the chief executive of the hospital trust. He showed his support for the study and the intervention through his regular e-newsletter sent to all staff, and through members of the Clinical Management Groups who were also asked to show support (ie, encourage involvement and allow time for intervention activities) and to filter this message down to the other management team leads.

*Environmental strategies*—After attendance at a seminar (see individual and group strategies for more information), participants were provided with a height adjustable desk or desk platform to enable them to sit or stand to work. They were given a choice between a full sized electric desk (twin leg single step stand desk 1200×800, MACOI, Kimbolton, UK), which allows the desk top to move up and down, or a choice of two sizes of desk platform (Pro Plus 30 or Pro Plus 48, VARIDESK; TX), which sits on the existing desk allowing the computer screen and keyboard to be moved up and down. This choice allowed flexibility for office set-up and to avoid testing the effectiveness of a specific type of desk rather than the height adjustable desk concept. We provided a brief training session on how to use the desk or platform and on the ergonomic set-up. A leaflet was also provided to reinforce these messages.

*Individual and group strategies*—An initial group based education seminar (around 30 minutes’ duration) was delivered, which covered the health consequences of sitting and the benefits of reducing and regularly breaking up sitting. These messages were also reinforced in a leaflet provided at the end of the seminar. Participants were given their baseline results from the activPAL device at the end of the seminar, which informed them of their sitting (total and prolonged), standing, and stepping time at work, and overall daily levels. They were then provided with an action plan and goal setting booklet and encouraged to set a goal around sitting less at work based on their activPAL feedback and to create an action plan for this to be achieved. We provided participants with a DARMA cushion (Darma, CA, USA). to enable them to more regularly track and self monitor their sitting time (total and prolonged) and be prompted (in the form of a vibration) to regularly break up sitting. This cushion, which can be placed on an office chair, is approximately 2.5 cm thick and uses Bluetooth to sync data with a mobile phone app to provide the participant with real-time feedback. The frequency of the vibration prompt is a user defined setting (eg, can be set up to vibrate every 30 or 45 minutes). Every few months the participants received posters, with either educational or motivational messages. To provide ongoing support to participants, a trained member of the research team offered brief (about 15 minutes) coaching sessions, either face-to-face or by telephone, at month 1 and every three months thereafter to discuss progress, review goals and action plans, and discuss personal or social and group barriers and any benefits experienced. After each visit for follow-up measurements, the participants were provided with their results from the activPAL device, and these were compared with the baseline data. This allowed the participants to review their progress and goals.

### Control group

Participants in control office clusters were not given any lifestyle advice, guidance, or results from the activPAL device. However, they received the results of health measures (eg, weight, blood pressure) taken at each time point (the intervention participants also received their own results). Other than this, these participants continued with usual practice for the 12 month study period.

### Statistical analysis

#### Sample size

After starting recruitment procedures, we amended our sample size calculation because of differences in office cluster sizes from our original plan. The study funder and sponsor agreed this amendment. The office cluster sizes were different because during the grant application process we approached managers within the hospital trust for their interest, and the original sample size was based on the department sizes of the managers who had expressed an interest in taking part. On commencement of the trial and advertising of the study, which was over two years after this initial contact, not all managers and staff within these initially identified potential clusters volunteered, but staff who were within other departments not originally identified did volunteer. These resulted in different clusters sizes. The published protocol^33^ outlines the original sample size of 238 participants from 14 clusters. The average cluster size was smaller than originally planned. After completion of recruitment, 37 office clusters were recruited, with an average office cluster size of 4 (range 1-16) office workers. This final sample size resulted in more than 90% power to detect a reduction of 60 min/workday (SD 60 min/workday^64^) in occupational sitting time between the groups, with a 25% drop-out and non-compliance to primary outcome taken into account. A 60 min/workday difference was chosen after consideration of the published literature at the time of designing the study.^1^ ^64^ ^65^

As with the initial sample size calculation this assumes an intraclass correlation coefficient of 0.05 and coefficient of variation for cluster size of 0.9. The sample size was robust to changes in the intraclass correlation coefficient—a value of 0.1 would still give over 90% power.

#### Data analysis

A statistical analysis plan was written, finalised, and agreed before data were available. We compared cluster and participant level characteristics by group allocation, using either means (standard deviations) or medians (interquartile ranges) for continuous variables, and counts and percentages for nominal variables.

The primary outcome, occupational sitting time (average min/workday) at 12 months, was analysed on a complete case basis using a generalised estimating equation model with an exchangeable correlation structure, accounting for clustering. The primary analysis was based on participants providing data for at least one valid workday from the activPAL device. The model included a binary indicator for randomisation group and was adjusted for baseline sitting time, cluster size (≤4 or >4 participants), and average activPAL wear time during work hours across baseline and 12 months.

We carried out several sensitivity analyses of the primary outcome and daily sitting time: intention to treat analysis with missing data imputed using multiple imputation,^66^ impact of variation in occupational or waking wear time, time spent in each activity, normalised to an eight hour workday and a 16 hour waking day as used in a previous similar study,^29^ and the effect of the number of valid activPAL working and overall days chosen for the primary analysis and how changing this affected the results. We assessed two scenarios: two working and overall days or more and three working and overall days or more.

To assess if the intervention effect was statistically different between groups we conducted several subgroup analyses: hospital site (Leicester General Hospital, Leicester Royal Infirmary, Glenfield General Hospital), worker status (part time, full time), sex (men, women), age (below or above the median), and body mass index (normal, overweight or obese (≥25 kg/m^2^). We included interaction terms in the generalised estimating equation models to assess differences between subgroups.

Secondary outcomes were also analysed using generalised estimating equation models with an exchangeable correlation structure (an independent structure was used where models did not converge). For binary outcomes we used a logit link with a binomial distribution for the outcome, and for continuous outcomes we used an identity link with a normal distribution. All primary and secondary analyses for the accelerometer (activPAL and ActiGraph) outcomes were adjusted for baseline value, office size, and average activPAL wear time during work hours (for occupational activPAL outcomes) and average activPAL waking wear hours (for daily activPAL outcomes) across baseline and outcome time. We repeated the analysis at each time point (3, 6, and 12 months). Adjustment for multiple testing of secondary outcomes was not performed. We interpreted outcomes according to the overall pattern of results; individual results should therefore be interpreted with caution. Statistical significance was set at 5%. All analyses were conducted using Stata version 14.

### Patient and public involvement

The public were involved in this study in several ways. Office workers within the target organisation contributed to the intervention strategies and content before they were developed. Lay members from within and outside the target organisation (NHS trust) sat on the trial steering committee. These members advised on practical issues such as logistics, space, and desk mechanics. Participants were invited to a presentation of results (two sessions offered at each hospital site), and an infographic of the results was designed and circulated to participants.

## Results

**Fig 1:**
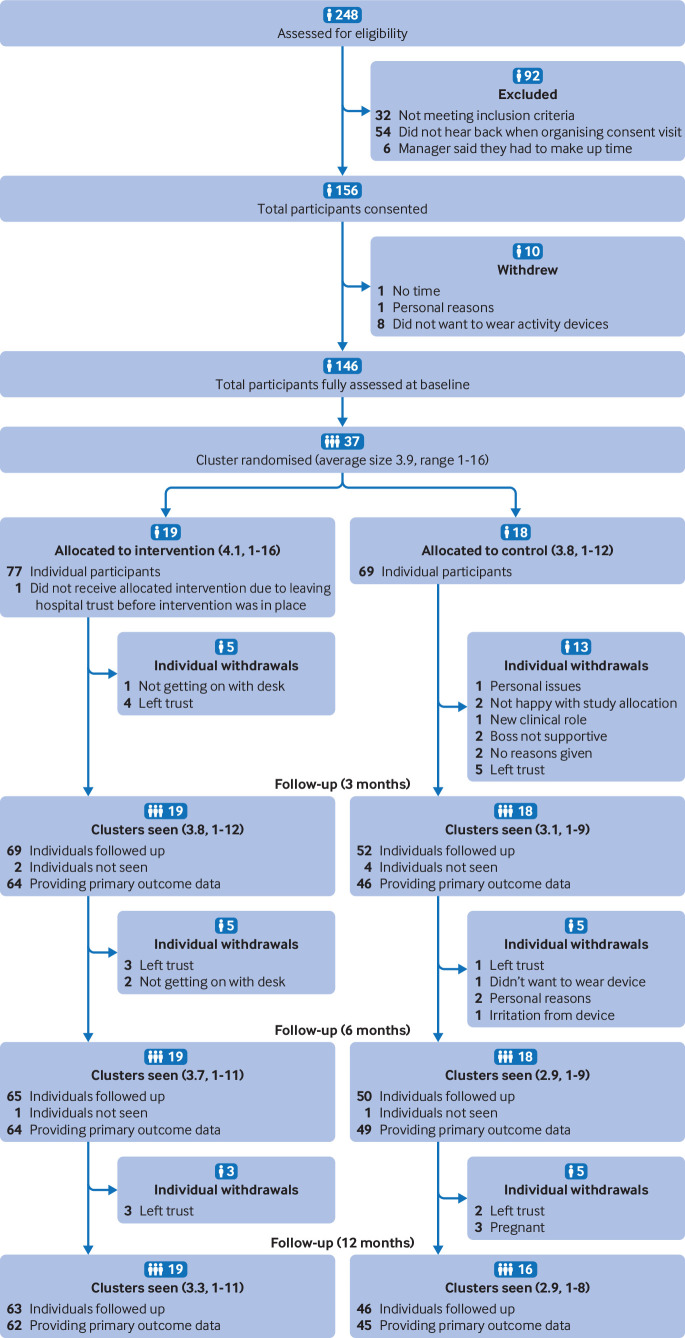
CONSORT flow diagram

Figure 1 displays the flow of participants through the study. Between November 2015 and June 2016, 146 participants across 37 office clusters were recruited, with 19 office clusters (77 participants) randomised to the intervention arm (one participant subsequently withdrew before intervention implementation, leaving 76 participants) and 18 clusters to the control arm (69 participants). Of these, 121 (83%), 115 (79%), and 109 (75%) participants and 100%, 100%, and 95% of clusters were seen at the 3, 6, and 12 month follow-up, respectively. More participants in the control group than intervention group withdrew from the study (control 33% *v* intervention 17%).

### Baseline characteristics

Table 1 presents the overall characteristics of the office clusters and the individual participants within these clusters. Office clusters ranged in size from one to 16 participants, with a mean of four participants in each cluster. The mean age of participants was 41.2 (SD 11.1) years, 78% reported being of white European ethnicity, and the majority were women (80%). Most of the participants (74%) worked full time and were spread across different NHS salary bands. On average, participants spent 72.6% (5.94 (SD 1.47) h/workday) of their work hours sitting, of which 47.1% (2.80 (1.60) h/workday) was accrued in prolonged bouts, 20.0% (3.84 (1.07) h/workday) was standing, and 7.5% (1.64 (0.29 h/workday) was stepping. Across daily waking hours, participants spent 63.7% (9.71 (1.55) h/day) of their day sitting, of which 51.4% (4.99 (1.75) h/day) was prolonged sitting, 25.3% (3.84 (1.34) h/day) standing, and 10.8% (1.64 (0.52) h/day) stepping. There were no significant differences between those with available primary outcome data at both baseline and 12 months and those without for the characteristics reported in table 1, except for salary banding (those on a higher salary were less likely to have available data). Participant characteristics of intervention and control participants were similar, except for ethnicity and sex. The intervention group consisted of more South Asian (21% *v* 13%) and more male (27% *v* 13%) participants than the control group. Of the participants randomised to the intervention, 40% (n=30) chose a full electric desk and 60% (n=46) chose the desk platform.

**Table 1:** Baseline characteristics at both cluster and individual levels according to randomised groups: usual practice (control) and SMArT Work intervention. Values are means (standard deviations) unless stated otherwise

Characteristics	Control	Intervention	Total
**Cluster level**	(n=18)	(n=19)	(n=37)
Mean No (range) of participants	4 (1-12)	4 (1-16)	4 (1-16)
Median No (interquartile range) of participants	2.5 (1.75-5.25)	3 (2-6)	3 (2-5.5)
Mean (range) proportion of cluster taking part (%)	44 (8-71)	41 (13-100)	43 (8-100)
No (%) of participants:			
≤4	12 (67)	14 (74)	26 (70)
>4	6 (33)	5 (26)	11 (30)
**Individual level**	(n=69)	(n=77)	(n=146)
Age (years)	40.8 (11.3)	41.7 (11.0)	41.2 (11.1))
No (%) of participants by site:			
Leicester General Hospital	14 (20)	26 (34)	40 (27)
Leicester Royal Infirmary	26 (38)	43 (56)	69 (47)
Glenfield Hospital	29 (42)	8 (10)	37 (25)
Ethnicity (No (%)):			
White European	57 (83)	57 (74)	114 (78)
South Asian	9 (13)	16 (21)	25 (17.2)
Other	3 (4)	3 (4)	6 (4.1)
No (%) women	60 (87)	56 (73)	116 (79)
No (%) men	9 (13)	21 (27)	30 (21)
Smoking status (No (%)):			
Current	5 (7)	3 (4)	8 (5)
Former	18 (26)	18 (23)	36 (25)
Never	46 (67)	56 (73)	102 (70)
Worker status (No (%)):			
Full time	51 (74)	57 (74)	108 (74)
Part time	18 (26)	20 (26)	38 (26)
Salary band (No (%))*:			
2-4	31 (45)	25 (32)	56 (38)
5-6	11 (16)	17 (22)	28 (19)
7-8	10 (14)	14 (18)	24 (16)
>8	4 (6)	8 (10)	12 (8)
Biometric measurements:			
Body mass index	26.7 (6.5)	25.8 (5.4)	26.2 (5.9)
Percentage body fat	31.9 (11.1)	29.0 (10.2)	30.4 (10.7)
Body weight (kg)	73.2 (19.2)	71.8 (15.2)	72.4 (17.2)
Waist circumference (cm)	86.5 (13.7)	85.5 (13.7)	86.0 (13.6)
Systolic blood pressure (mm Hg)	118.3 (15.9)	119.8 (11.3)	119.1 (13.6)
Diastolic blood pressure (mm Hg)	82.6 (12.2)	81.3 (9.1)	81.9 (10.6)
**activPAL and ActiGraph variables**			
Overall daily values:			
Sitting (min/day)	584.4 (94.4)	580.9 (91.9)	582.6 (92.8)
Prolonged (≥30 mins) sitting (min/day)	300.1 (108.4)	298.4 (102.2)	299.2 (104.8)
Standing (min/day)	225.4 (82.7)	234.8 (78.9)	230.4 (80.6)
Stepping (min/day)	93.7 (29.4)	102.1 (32.8)	98.1 (31.4)
Wear time (min/day)	903.6 (54.6)	917.9 (51.3)	911.1 (53.2)
No of valid days	6.2 (1.4)	6.5 (1.2)	6.3 (1.3)
MVPA (ActiGraph) (min/day)	88.40 (31.29)	96.81 (42.27)	92.90 (37.68)
No of valid days (ActiGraph)	6.09 (1.16)	5.61 (1.36)	5.83 (1.29)
Occupational values:			
Sitting (min/day)	354.1 (90.5)	357.9 (86.6)	356.1 (88.2)
Prolonged (≥30 mins) sitting (min/day)	168.8 (100.5)	166.7 (92.3)	167.7 (95.9)
Standing (min/day)	104.2 (74.0)	92.7 (53.9)	98.2 (64.2)
Stepping (min/day)	36.8 (18.1)	37.0 (17.2)	36.9 (17.6)
Wear time (min/day)	493.7 (69.9)	487.2 (62.7)	490.3 (66.1)
No of valid work days	4.1 (1.0)	4.0 (1.1)	4.1 (1.1)
MVPA (ActiGraph) (min/day)	34.93 (18.49)	34.84 (19.80)	34.88 (19.14)
No of valid days (ActiGraph)	3.68 (1.16)	3.38 (1.20)	3.52 (1.19)

MVPA=moderate to vigorous physical activity.

* Higher number represents higher salary.

### Change in occupational sitting time at 12 months (primary outcome)

Table 2 reports the mean change in occupational sitting time by randomisation group and the difference in change between groups at 12 month follow-up. In the complete case analysis, a statistically significant difference between groups was found in occupational sitting time (adjusted difference −83.28 min/workday, 95% confidence interval −116.57 to −49.98 min/workday) in favour of the intervention group. Similar results were seen in the intention to treat analysis (table 2).

**Table 2:** Changes in occupational sitting time at 12 month follow up between participants randomised to usual practice (control) or SMArT Work intervention

Variables	No of clusters (participants)			Mean change from baseline (95% CI)			Adjusted difference at follow-up*	
	Control	Intervention		Control	Intervention		Coefficient (95% CI)	P value
**Complete case†**								
Occupational sitting (min/workday)	16 (45)	19 (62)		9.22 (−17.65 to 36.09)	−71.99 (−97.37 to −46.61)		−83.28 (−116.57 to −49.98)	0.001
**Intention to treat‡**								
Occupational sitting (min/workday)	18 (69)	19 (77)		6.59 (−20.46 to 33.64)	−77.58 (−101.62 to −53.54)		−81.64§ (−112.27 to −51.01)	<0.001
**Standardised waking/occupational hours**								
8 hour workday¶:								
Occupational sitting (min/8 h workday)	16 (45)	19 (62)		3.78 (−11.23 to 18.78)	−35.21 (−49.12 to −21.31)		−41.29 (−59.88 to −22.69)	<0.001
**Effect on No of valid activPAL days**								
≥2 working days:								
Occupational sitting (min/workday)	15 (44)	19 (60)		11.19 (−16.01 to 38.39)	−73 (−99.63 to −47.92)		−86.14 (−119.38 to −52.90)	<0.001
≥3 working days:								
Occupational sitting (min/workday)	15 (41)	17 (52)		6.45 (−21.28 to 34.18)	−74.06 (−102.78 to −45.35)		−80.66 (−111.74 to −49.58)	<0.001

* Adjusted difference in mean sitting time at follow-up between treatment groups, P value adjusted for cluster effect, baseline sitting time, average activPAL wear time during work hours across baseline and 12 months and stratification categories (cluster size ≤4 and >4 participants).

† Including participants who have worn the accelerometer with a minimum of one valid day at baseline and 12 months.

‡ Missing data imputed using multiple imputation.

§ Convergence not achieved—independent correlation structure used.

¶ Averaged over amount of time (in hours) participants wore device and normalised to 8 hour workday.

Sensitivity analyses (table 2) showed similar results to the primary analysis for occupational sitting time, with statistically significant differences between groups at 12 months when the various levels of activPAL data were used (ie, including only those with at least two and three valid days). Although a significant difference between groups for occupational sitting was found when standardising the data to an eight hour workday, the difference was smaller (−41.29 min/8 h workday).

**Fig 2:**
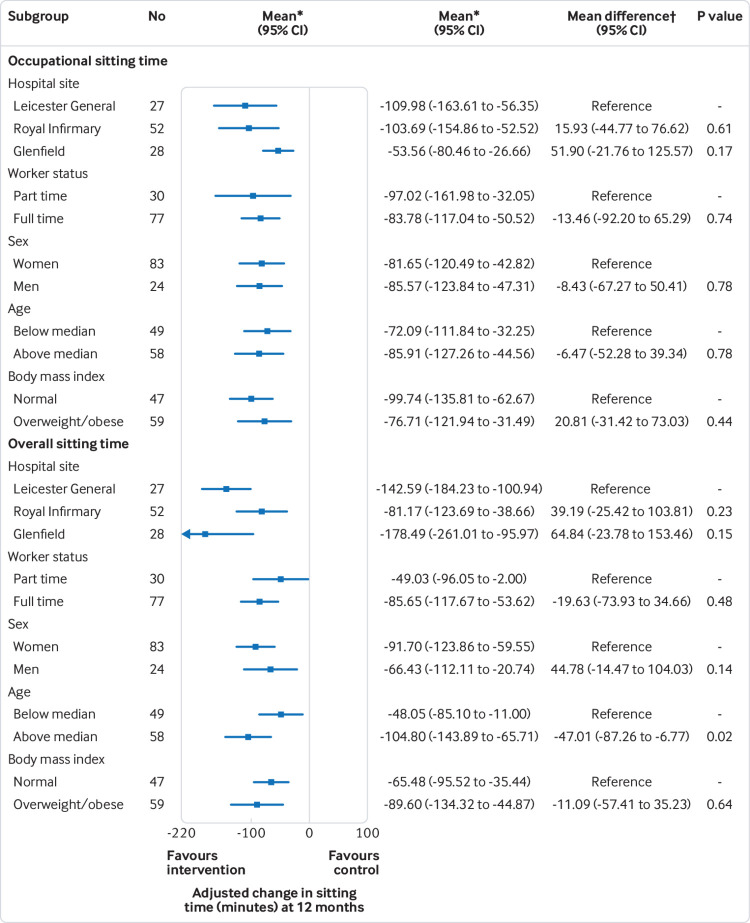
Forest plot of intervention effect at 12 months on occupational and daily sitting time by subgroup. *Adjusted for cluster effect, baseline occupational sitting, baseline overall sitting, stratification category (office size ≤4 or >4 participants) and average activPAL wear times during work hours/average activPAL waking wear time across baseline and 12 months. †Interaction between intervention group and subgroups

Figure 2 shows the results of the subgroup analyses. No statistically significant interaction effects were found for change in occupational sitting time.

### Secondary outcomes

#### activPAL and ActiGraph outcomes

Table 3 presents the secondary outcomes collected by the activPAL and ActiGraph. Differences between groups were found in occupational sitting time at three months (−50.62 min/workday) and six months (−64.40 min/workday) and daily sitting time at six (−59.32 min/workday) and 12 months (−82.39 min/workday) in favour of the intervention group compared with control, indicating that the intervention group spent significantly less time sitting than the control group. Similar results for daily sitting time at 12 months were seen in the intention to treat analysis, when standardising the daily sitting time data to a 16 hour waking day and when the various levels of activPAL data were used (ie, including only those with at least two and three valid days) (data not shown). Figure 2 displays the results of the subgroup analyses for daily sitting time at 12 months. For most subgroups there were no interaction effects. However, an interaction effect was found for age. At 12 months, the between subgroup interaction effects (P=0.02) revealed that the intervention was more effective for participants above the median age (42.5 years), who reduced their daily sitting by 45.11 additional minutes daily than those below the median age.

**Table 3:** Changes in secondary outcome sitting and physical activity variables at follow-up between participants randomised to usual practice (control) or to the SMArT Work intervention*

Secondary outcomes	No of offices (participants)			Mean change from baseline (95% CI)			Adjusted difference at follow-up†	
	Control	Intervention		Control	Intervention		Coefficient (95% CI)	P value
**Occupational sitting time (min/workday)**								
3 months	17 (46)	19 (63)		−6.17 (−30.24 to 17.91)	−62.09 (−83.89 to −40.30)		−50.62 (−78.71 to −22.54)	<0.001
6 months	18 (48)	19 (64)		−0.25 (−26.17 to 25.67)	−61.50 (−86.47 to −36.53)		−64.40 (−97.31 to −31.50)	<0.001
**Daily sitting time (min/day)**								
3 months	17 (46)	19 (64)		−27.57 (−58.6 to 3.43)	−49.01 (−69.41 to −28.61)		−33.46 (−67.24 to 0.32)	0.05
6 months	18 (49)	19 (64)		4.84 (−24.80 to 34.49)	−42.38 (−64.43 to −20.33)		−59.32 (−88.40 to −30.25)	<0.001
12 months	16 (45)	19 (62)		7.99 (−17.17 to 33.14)	−63.03 (−86.01 to −40.04)		−82.39 (−114.54 to −50.26)	0.001
**Occupational prolonged sitting time (min/workday)**								
3 months	17 (46)	19 (63)		–13.73 (−34.59 to 7.12)	−35.77 (−54.83 to −16.71)		−17.49 (−44.27 to 9.29)	0.20
6 months	18 (48)	19 (64)		−5.12 (−30.09 to 19.86)	−40.81 (−60.08 to −21.54)		−35.31 (−58.75 to −11.87)	0.003
12 months	16 (45)	19 (62)		3.87 (−24.38 to 32.13)	−36.84 (−59.80 to −13.88)		−44.93 (−79.67 to −10.20)	0.011
**Daily prolonged sitting time (min/day)**								
3 months	17 (46)	19 (64)		−32.19 (−62.56 to −1.81)	−41.24 (−61.78 to −20.71)		−12.33 (−29.88 to 5.23)	0.17
6 months	18 (49)	19 (64)		1.50 (−24.36 to 27.36)	−32.35 (−50.83 to −13.87)		−25.38 (−44.66 to −6.10)	0.010
12 months	16 (45)	19 (62)		10.90 (−17.01 to 38.82)	−37.19 (−59.45 to −14.93)		−58.34 (−91.18 to −25.50)	<0.001
**Occupational standing time (min/workday)**								
3 months	17 (46)	19 (63)		7.99 (−16.93 to 32.92)	58.43 (38.18 to 78.67)		48.91 (19.21 to 78.61)	<0.001
6 months	18 (48)	19 (64)		−3.94 (−27.15 to 19.28)	68.34 (46.59 to 90.09)		72.62 (44.80 to 100.44)	<0.001
12 months	16 (45)	19 (62)		−4.15 (−28.89 to 20.59)	66.92 (46.04 to 87.80)		66.00 (38.14 to 93.86)	<0.001
**Daily standing time (min/day)**								
3 months	17 (46)	19 (64)		14.40 (−8.97 to 37.78)	44.45 (27.35 to 61.53)		36.95 (9.28 to 64.62)	0.009
6 months	18 (49)	19 (64)		−7.48 (−31.73 to 16.66)	43.28 (23.39 to 63.18)		55.96 (28.23 to 83.69)	<0.001
12 months	16 (45)	19 (62)		−6.31 (−28.46 to 15.85)	49.38 (29.08 to 69.67)		62.81 (36.24 to 89.39)	<0.001
**Occupational stepping time (min/workday)**								
3 months	17 (46)	19 (63)		1.28 (−2.51 to 5.07)	2.41 (−1.57 to 6.39)		1.33‡ (−2.68 to 5.34)	0.52
6 months	18 (48)	19 (64)		2.26 (−2.13 to 6.65)	4.56 (0.59 to 8.53)		3.56 (−1.18 to 8.30)	0.14
12 months	16 (45)	19 (62)		0.33 (−4.07 to 4.73)	−1.61 (−5.70 to 2.48)		0.0003 (−5.15 to 5.15)	1.00
**Daily stepping time (min/day)**								
3 months (min/day)	17 (46)	19 (64)		15.95 (8.26 to 23.64)	8.69 (3.08 to 14.30)		−6.44 (−16.16 to 3.28)	0.19
6 months (min/day)	18 (49)	19 (64)		1.29 (−5.76 to 8.35)	5.11 (−0.53 to 10.76)		7.06 (−2.39 to 16.50)	0.14
12 months (min/day)	16 (45)	19 (62)		3.75 (−2.81 to 10.32)	2.63 (−3.35 to 8.61)		1.21 (−8.18 to 10.60)	0.80
**Occupational MVPA (ActiGraph) (min/workday)**								
3 months	17 (43)	19 (61)		3.71 (−4.63 to 12.05)	0.50 (−3.51 to 4.51)		−1.72 (−6.68 to 3.23)	0.50
6 months	15 (38)	18 (57)		3.04 (−1.58 to 7.66)	1.39 (−2.63 to 5.40)		−0.61 (−7.89 to 6.68)	0.87
12 months	16 (38)	19 (56)		0.12 (−4.04 to 4.30)	−0.15 (−4.96 to 4.66)		2.26 (−4.22 to 8.74)	0.49
**Daily MVPA (ActiGraph) (min/day)**								
3 months	17 (45)	19 (64)		10.48 (1.11 to 19.85)	−0.38 (−6.22 to 5.46)		−9.75 (−20.86 to 1.36)	0.09
6 months	15 (41)	18 (57)		0.44 (−7.88 to 8.76)	1.43 (−5.02 to 7.88)		4.50 (−6.64 to 15.64)	0.43
12 months	16 (39)	19 (56)		0.87 (−7.60 to 9.34)	0.46 (−7.01 to 7.92)		1.01 (−9.27 to 11.29)	0.85

MVPA=moderate to vigorous physical activity.

* Including participants who have worn the accelerometer with a minimum of one valid day at baseline and three months, baseline and six months, and baseline and 12 months.

† Adjusted difference in mean sitting at follow-up between treatment groups with 95% confidence interval, P value; adjusted for cluster effect, baseline sitting, average activPAL wear time during work hours/activPAL waking wear time across baseline and three months, average activPAL wear time during work hours/activPAL waking wear time across baseline and six months, average activPAL wear time during work hours/activPAL waking wear time across baseline and 12 months and stratification categories (cluster size ≤4 and >4 participants).

‡ Used independent correlation structure. Model did not converge with exchangeable correlation structure.

Differences were found between groups in prolonged sitting time at six months (occupational: −35.31 min/workday, daily: −25.38 min/day) and 12 months (occupational: −44.93 min/workday, daily: −58.34 min/day) in favour of the intervention group compared with control, but not at three months.

The intervention group stood more than the control group at all time points, with group differences in occupational standing of 48.91, 72.62, and 66.00 min/workday, respectively, and in daily standing of 36.95, 55.96, and 62.81 min/day, respectively. No differences were found in occupational or daily stepping time and moderate to vigorous physical activity at any time point as measured by the ActiGraph (P>0.05).

#### Musculoskeletal problems

At baseline, a high proportion of participants in both groups reported experiencing musculoskeletal problems in the previous 12 months (see supplementary table 2). No differences were found between groups at the 12 month follow-up in the proportion of participants reporting musculoskeletal problems (neck, lower back, upper extremity, lower extremity, any part) and the pain experienced from musculoskeletal problems in the previous 12 months (P>0.05). A difference between groups was, however, found for the proportion of participants reporting that lower back problems prevented them from carrying out normal activities, with the odds of lower back problems preventing them from carrying out normal activities being less in the intervention group. Differences between groups were also found for musculoskeletal problems reported over the past seven days for the neck and upper extremity areas at 12 month follow-up and any part at six months, with the odds of reporting problems being less in the intervention group.

#### Work related outcomes

*Work engagement*—Differences (in favour of the intervention group versus control) at six and 12 months were observed for the vigour subscale and for overall work engagement (see supplementary table 3). Differences at 12 months (in favour of the intervention group) were seen for work dedication and work absorption. No differences were found at three months.

*Job satisfaction and performance and occupational fatigue*—Differences at six and 12 months (in favour of the intervention group) were observed in job performance and recovery from occupational fatigue, but not in job satisfaction. No differences were found at three months.

*Sickness presenteeism*—Differences were observed between groups, in favour of the intervention group compared with control, in the scales of time management and mental-interpersonal demands and for overall sickness presenteeism at 12 and three months, respectively.

*Sickness absence*—No differences between groups were seen for either self reported or organisation reported (see supplementary table 4) sickness absence from work (P>0.05).

#### Cognitive function outcomes

Supplementary table 5 displays the results for the cognitive function tests. There were differences between groups in reaction times at 3, 6, and 12 months for the congruent level of the Stroop Colour-Word Test and in proportion of correct hits at the incongruent level, all in favour of the intervention group compared with control.

#### Mood, mental health, and quality of life

For most mood affect variables no differences were observed between groups (see supplementary table 6). However, differences were found for anxiety today at six and 12 months and dysphoria today at six months, in favour of the intervention compared with control.

Between group differences were found for anxiety generally at three months, hostility generally at 12 months, and dysphoria generally at three months, in favour of the control group.

Quality of life was assessed in four individual domains and overall (see supplementary table 7). Between group differences were found in two domains of quality of life and for the overall score at six and 12 months, all in favour of the intervention group compared with control. Participants in the intervention group compared with control group reported an improvement in their psychological, environmental, and overall quality of life.

## Discussion

This cluster randomised controlled trial evaluated the effectiveness of a multicomponent intervention, involving a height adjustable workstation, for reducing occupational sitting time in a sample of office workers based within the University Hospitals of Leicester NHS Trust. The SMArT Work intervention resulted in reductions in occupational and daily sitting time over the short (three months), medium (six months), and longer term (12 months). The reduction in sitting was largely replaced by time spent standing, as stepping time remained unchanged. Although a reduction in daily sitting time was observed, this was of a similar magnitude to the reduction seen during work time, suggesting that the changes seen for daily sitting time were likely due to changes made at work. Time spent in prolonged sitting was also reduced in the intervention group. Results were also suggestive of improvements and benefits in assessed secondary outcomes, including job performance, work engagement, occupational fatigue, sickness presenteeism, and psychological health, although these tended to be at the later follow-up time points. No notable changes were found in job satisfaction, cognitive function, and sickness absence.

### Comparison with other studies

The majority of previous workplace interventions employing height adjustable workstations have been evaluated over the short term (eg, three months) using small samples, and observed sitting reductions of between 30 minutes and two hours daily,^25^ which is comparable with the present study. Other recent larger studies, evaluating similar multicomponent interventions, have also exhibited similar behaviour changes.^29^ ^67^ However, although these studies observed reductions at their concluding assessment time point, these tended to be smaller than those observed at the shorter term follow-up. In the present study, the reductions in sitting at three months were not only maintained at both subsequent follow-up time points (six and 12 months) but were largest at the final follow-up assessment at 12 months. We included a six month follow-up assessment where participants received feedback on their health and behaviour, and one-to-one coaching was continued throughout the whole study period. This may indicate that the ongoing coaching sessions or feedback on health and behaviour, or both, were able to assist the participants in maintaining their initial behaviour change. The value of regular contact was highlighted as a motivating factor in the process evaluation focus groups. A previous study targeting sitting also highlighted that regular assessments motivated participants.^68^

Consistent with previous research,^26^ ^29^ ^67^ sitting was replaced with standing rather than ambulation, despite emphasis on both behaviours. Participants may have chosen to reduce their sitting time by performing work tasks standing at their desk rather than reducing and breaking up their sitting through activities such as using a toilet, printer, or water cooler further away, walking meetings, or a combination of both strategies, suggestions that were promoted in the intervention. More qualitative research may be needed to elicit how best to encourage changes in movement while at work, in terms of the ability to perform work tasks more actively and to incorporate more movement during work breaks.

Participants in the intervention group on average reduced their sitting time by more than an hour daily (95% confidence interval of 40 to >85 min/day reduction in sitting), and a recent meta-analysis examining the strength and shape of the dose-response relation between sedentary behaviour and health outcomes, suggests that this may have meaningful health benefits.^69^ For example, the increased risk of all cause and cardiovascular mortality was strongest for those sitting for more than 8 h/day (relative risk of 1.04 for each additional hour after eight hours) and 6 h/day (relative risk of 1.04 for each additional hour after six hours), respectively. The average daily sitting time of the intervention group at baseline was 9.7 h/day. Furthermore, the association between sitting time and type 2 diabetes appeared to be linear, suggesting that any reduction may be beneficial. However, the acute experimental evidence is equivocal for replacing sitting time with standing time and the resulting metabolic health benefits, with one study showing that breaking up sitting with standing has acute beneficial effects on postprandial metabolic health in those with impaired glucose regulation,^15^ with other studies reporting a modest^16^ ^70^ or no effect^19^ ^71^ in healthy populations. Future studies are therefore needed to assess the benefit of displacing sitting with standing on health outcomes over the longer term. Nevertheless, an increase in standing seemed to have a positive impact on many work related outcomes such as job performance, work engagement, occupational fatigue, sickness presenteesim, and some musculoskeletal problems. In previous similar shorter term (eg, three months) interventions, self reported or objectively measured work performance were not negatively or positively affected.^25^ ^26^ Our findings suggest that this type of intervention may take time to positively affect work performance, as these differences were observed in the present study at six and 12 months.

While levels of sickness absence across the UK have remained relatively stable (6.6 days per person in 2014 and 6.9 days per person in 2015), presenteeism is a growing problem for employers. Consequently, presenteeism is now more costly than sickness absenteeism, £21.2 bn per annum versus £10.6 bn per annum, respectively.^72^ Work engagement is an important indicator of productivity, turnover, and wellbeing of the workforce,^46^ ^73^ and occupational fatigue has been associated with unintentional injuries at work^74^ and health problems,^75^ ^76^ while both have been linked to sickness absenteeism.^75^ ^77^ ^78^ Despite positive changes to work engagement (all subscales at 12 months and overall work engagement) and occupational fatigue, we did not observe any differences in self reported or organisational records of sickness absence. Given that the positive changes observed in other work related outcomes occurred later in the randomised controlled trial (six and 12 months), any impact on sickness absenteeism may emerge in future months. Nevertheless, positive changes in sickness presenteeism were found for the domains of time management and mental-interpersonal demands, when measured using the Work Limitations Questionnaire.

Recent data reported that half a million employees experienced work related musculoskeletal disorders in Great Britain in 2016-17, which resulted in 8.9 million working days lost.^79^ We also observed a high prevalence of musculoskeletal conditions in this office based sample and although we did not find any differences reported over the whole 12 month randomised controlled trial period, the prevalence of neck and upper extremity problems experienced in the past seven days at 12 months, and the proportion of lower back problems interfering with normal activities, was lower in the intervention group. Results from previous research with similar interventions have been mixed in terms of the benefits for musculoskeletal problems. One study reported a non-significant increase in musculoskeletal conditions,^8^ several studies reported no differences,^26^ ^80^ ^81^ whereas other studies have reported slight decreases in lower back pain,^82^ upper back pain,^65^ and neck pain.^65^ One recent review concluded that sit-stand workstations may help reduce low back pain in workers.^83^

A small body of epidemiological evidence suggests that lower levels of sedentary behaviour are associated with higher quality of life scores.^84^ ^85^ Our results corroborate these findings, with increases in quality of life reported by the intervention participants.

Taking these findings together, this type of intervention (providing an environmental change combined with additional strategies such as education, self monitoring, and brief coaching) may be of benefit to employers in terms of having more engaged and higher performing staff as well as cost saving from sickness presenteeism, musculoskeletal problems, and potentially sickness absenteeism. A separate paper will formally assess the cost effectiveness of the intervention.

### Strengths and limitations of this study

The strengths of this study include the robust randomised controlled design, with randomisation at the cluster level, the fully powered sample size, the short, medium, and longer term follow up assessments, and the device based measurement of the primary outcome. Therefore, this study tackles many of the limitations of previous evaluations of workplace interventions focused on reducing sitting time.^25^ Furthermore, we performed several sensitivity analyses to check the robustness of our results. Although the study had a 27% loss to follow-up/non-compliance with primary outcome assessment by 12 months, our sample size was sufficiently large enough to account for this drop-out. This drop-out/non-compliance rate is similar to that seen at 12 months in the Stand Up Victoria study.^29^ The conduct of the present study in an NHS trust is both a strength and a limitation. The NHS is the fifth largest employer globally, with around 1.3 million staff. Clerical and administrative staff make up about a third of NHS employees, therefore this intervention has potential to reach a large number of people. Conversely, as the study was only conducted in a single organisation this may limit the generalisibility of the intervention and findings to other types of organisations beyond the NHS, particularly those with large open plan offices, which were rare within the University Hospitals of Leicester NHS Trust. Although we used an objective assessment of sitting time and physical activity and removed the first day of data collection from the activPAL, it is possible that reactivity (change in behaviour from an awareness of being monitoring) may have biased the results. Many of our work related outcomes were assessed by self report and may have been subject to reporting bias. As SMArT Work was a complex intervention it had the potential to exert effects at many levels, therefore we included many outcomes. However, this study was not powered to detect differences in all of the measured outcomes, and adjustment for multiple comparisons was not performed. The emphasis therefore should be on the pattern of the secondary outcome results.

### Conclusions

The SMArT Work multicomponent intervention was able to reduce occupational and daily sitting time in the short, medium, and longer term in office workers within the University Hospitals of Leicester NHS Trust. The intervention also appeared to have a positive impact on musculoskeletal conditions and many work related outcomes such as job performance, work engagement, occupational fatigue, and sickness presenteeism as well as being beneficial for psychological outcomes such as daily anxiety and quality of life. Areas for future research include the replication of these findings in other organisations, focusing interventions on standing and moving more throughout the whole day (ie, taking a whole day approach to reductions in sitting), eliciting how best to promote movement rather than just standing, and longer term follow up to assess maintenance of behaviour change and allow sufficient time to impact those outcomes that take longer to influence, such as absenteeism.

**What is already known on this topic**

High levels of sedentary behaviour (sitting) have been associated with an increased risk of morbidity and mortality and have been shown to be detrimental for work related outcomes such as engagement and presenteeism
Office workers are one of the most sedentary populations, spending 70-85% of time at work sitting
Interventions to reduce sitting in the workplace have received increasing attention in recent years but studies to evaluate these have been deemed low quality

**What this study adds**

The SMArT Work multicomponent intervention involving a height adjustable workstation, successfully reduced occupational sitting time over the short, medium, and longer term in a sample of office workers
Positive changes were observed in work related and psychological health

## Web extra

Extra material supplied by authors

**Supplementary information**: Logic model

## Notes

Contributors: CLE, SJHB, MJD, DWD, DWE, LJG, TY, and FM obtained funding for the research. All authors have contributed to the design of the study. GW performed the statistical analysis, supervised by LJG. BJ was involved in data collection at the start of the project and SEO’C was involved in data collection and study coordination throughout. CLE and FM supervised BJ and SOC. CLE processed the activPAL data. The first draft of this manuscript was produced by CLE and all authors have reviewed, edited, and approved the final version. CLE and FM are the guarantors. The corresponding author attests that all listed authors meet authorship criteria and that no others meeting the criteria have been omitted.

Ethical approval: This study was approved by Loughborough University, and Research and Innovation approval was obtained from the University Hospitals of Leicester NHS Trust (EDGE ID 34571).

Data sharing: Requests for access to data from the study should be addressed to the corresponding author at ce95@le.ac.uk. The study protocol has been published. All proposals requesting data access will need to specify how it is planned to use the data, and all proposals will need approval of the trial co-investigator team before data release.

Transparency: The guarantors (CLE, FM) affirm that the manuscript is an honest, accurate, and transparent account of the study bring reported; that no important aspects of the study have been omitted; and that any discrepancies from the study as planned have been explained.
