# Supplementary figures and images for: Effectiveness of the Stand More AT (SMArT) Work intervention: cluster randomised controlled trial
Source: BMJ. 2018 Oct 10;363:k3870. doi: 10.1136/bmj.k3870 (PMC6174726; doi:10.1136/bmj.k3870)

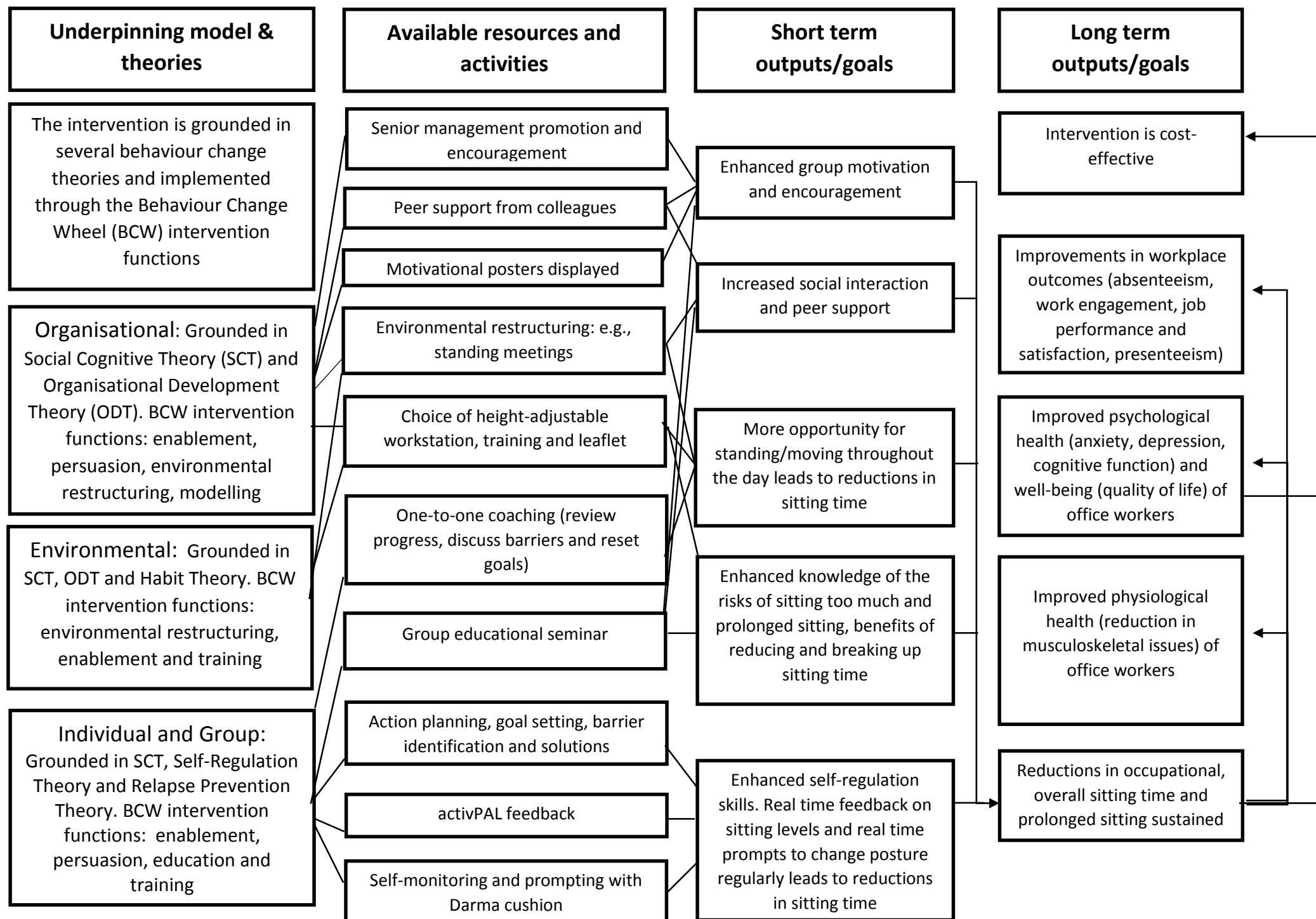

Supplement: Supplementary file 1 — Supplementary information: Logic model [file edwc044237.wf1.pdf]
