# Supplementary material for: Effectiveness of the Stand More AT (SMArT) Work intervention: cluster randomised controlled trial
Source: BMJ. 2018 Oct 10;363:k3870. doi: 10.1136/bmj.k3870 (PMC6174726; doi:10.1136/bmj.k3870)
Supplement: Supplementary file 2 — Supplementary information: additional tables 1-7 [file edwc044237.wt1.pdf]

Supplementary Table 1. Intervention timeline

Intervention	Level of intervention	Baseline	Intervention months											
			1	2	3	4	5	6	7	8	9	10	11	12
Provision of height-adjustable workstation	Environmental													
Educational seminar	Group													
Demonstration of desk	Individual													
Office-based delivery	Social													
Leaflets	Individual													
Posters*	Organisational													
Diary	Individual													
Darma Cushion	Individual													
Coaching session	Individual													
ActivPAL feedback on sitting time (after each data collection)	Individual													

Supplementary Table 2 Changes in musculoskeletal issues at follow up for participants randomised to usual practice (control) or to the SMArT Work intervention

	Number of offices (participants)		Mean change from baseline (95% CI)		Adjusted difference at follow-up [a]	
	Control	Intervention	Control	Intervention	Coefficient (95% CI)	P value
**Experience of pain in the last 12 months [b]**						

Neck	8 (10)	15 (19)	−0.80 (−2.37 to 0.77)	−0.47 (−1.10 to 0.16)	0.11 (−1.06 to 1.27)	0.86
Lower back	13 (21)	15 (23)	−0.81 (−2.03 to 0.42)	−1.09 (−2.13 to −0.04)	−0.07 (−1.53 to 1.39)	0.93
Upper extremity	12 (20)	15 (29)	−0.33 (−1.06 to 0.41)	−0.58 (−0.94 to −0.21)	−0.33* (−0.80 to 0.14)	0.16
Lower extremity	12 (17)	16 (21)	−0.75 (−1.50 to 0.01)	−0.44 (−1.11 to 0.22)	0.34 (−0.40 to 1.09)	0.37
Any part	16 (33)	18 (42)	−0.42 (−0.77 to −0.07)	−0.58 (−0.84 to −0.33)	−0.12 (−0.49 to 0.24)	0.50
	**Control**	**Intervention**	**Control**	**Intervention**	**Odds ratio (95%CI)**	**P value**
**Proportion of musculoskeletal issues in last 12 months** [c]						
Neck						
Baseline	18 (62)	19 (72)	0.50 (0.38 to 0.62)	0.54 (0.42 to 0.65)	-	
12 months	15 (35)	17 (51)	0.34 (0.20 to 0.52)	0.47 (0.34 to 0.61)	1.17 (0.41 to 3.29)	0.77
Lower back						
Baseline	18 (63)	19 (75)	0.70 (0.57 to 0.80)	0.67 (0.55 to 0.76)	-	
12 months	15 (39)	18 (54)	0.64 (0.48 to 0.79)	0.44 (0.32 to 0.58)	0.46 (0.19 to 1.14)	0.09
Upper extremity						
Baseline	18 (61)	19 (74)	0.69 (0.56 to 0.79)	0.70 (0.59 to 0.80)	-	
12 months	15 (38)	17 (56)	0.60 (0.44 to 0.75)	0.61(0.47 to 0.73)	1.33 (0.50 to 3.54)	0.57
Lower extremity						
Baseline	18 (62)	18 (71)	0.61 (0.48 to 0.73)	0.54 (0.42 to 0.65)	-	
12 months	16 (40)	18 (52)	0.50 (0.35 to 0.65)	0.48 (0.35 to 0.62)	1.47 (0.62 to 3.49)	0.39
Any part						
Baseline	18 (66)	19 (77)	0.89 (0.79 to 0.95)	0.87 (0.77 to 0.93)	-	
12 months	16 (42)	19 (59)	0.81 (0.66 to 0.90)	0.75 (0.62 to 0.84)	0.84 (0.35 to 2.01)	0.69
**Prevent from normal activities in the last 12 months due to:**						
Neck						
Baseline	14 (30)	15 (36)	0.17 (0.07 to 0.35)	0.14 (0.06 to 0.30)	-	
12 months	7 (11)	15 (23)	0.27 (0.08 to 0.61)	0.04 (0.005 to 0.27)	0.13 (0.01 to 1.93)	0.14
Lower back						
Baseline	15 (44)	17 (48)	0.43 (0.29 to 0.58)	0.33 (0.21 to 0.48)	-	
12 months	12 (24)	15 (23)	0.50 (0.30 to 0.70)	0.26 (0.12 to 0.48)	0.16 (0.04 to 0.69)	**0.01**
Upper extremity						
Baseline	17 (40)	17 (49)	0.27 (0.16 to 0.44)	0.24 (0.14 to 0.39)	-	
12 months	12 (23)	15 (33)	0.26 (0.12 to 0.48)	0.24 (0.12 to 0.42)	1.01 (0.19 to 5.38)	0.99
Lower extremity						
Baseline	16 (37)	17 (37)	0.30 (0.17 to 0.47)	0.40 (0.26 to 0.57)	-	

12 months	12 (19)	16 (25)	0.42 (0.22 to 0.66)	0.40 (0.22 to 0.61)	0.37 (0.12 to 1.11)	0.08
Any part						
Baseline	18 (59)	19 (67)	0.49 (0.36 to 0.62)	0.37 (0.27 to 0.49)	-	
12 months	16 (34)	18 (44)	0.50 (0.33 to 0.67)	0.36 (0.23 to 0.52)	0.38 (0.12 to 1.16)	0.09
**Trouble in the last seven days in:** [b]						
Neck						
Baseline	14 (29)	16 (38)	0.38 (0.22 to 0.57)	0.47 (0.32 to 0.64)	-	
3 months	11 (18)	14 (26)	0.33 (0.15 to 0.59)	0.50 (0.31 to 0.69)	1.41* (0.39 to 5.16)	0.60
6 months	10 (17)	14 (22)	0.53 (0.29 to 0.76)	0.46 (0.26 to 0.67)	0.90 (0.13 to 6.32)	0.92
12 months	7 (11)	15 (23)	0.46 (0.19 to 0.75)	0.26 (0.17 to 0.49)	0.03 (0.002 to 0.35)	**0.006**
Upper extremity						
Baseline	17 (37)	17 (50)	0.59 (0.43 to 0.74)	0.44 (0.31 to 0.58)		
3 months	12 (22)	14 (36)	0.36 (0.19 to 0.59)	0.42 (0.26 to 0.59)	1.56* (0.62 to 3.95)	0.35
6 months	14 (28)	17 (37)	0.57 (0.38 to 0.74)	0.43 (0.28 to 0.60)	0.56 (0.18 to 1.72)	0.31
12 months	11 (21)	15 (33)	0.62 (0.39 to 0.80)	0.27 (0.14 TO 0.45)	0.14 (0.03 TO 0.68)	**0.01**
Lower extremity						
Baseline	16 (37)	17 (37)	0.51 (0.35 to 0.67)	0.65 (0.48 to 0.79)	-	
3 months	14 (23)	14 (28)	0.61 (0.40 to 0.79)	0.50 (0.32 to 0.68)	0.64 (0.15 to 2.63)	0.53
6 months	13 (24)	17 (27)	0.71 (0.49 to 0.86)	0.67 (0.46 to 0.82)	0.56 (0.20 to 1.57)	0.27
12 months	12 (20)	16 (24)	0.65 (0.41 to 0.83)	0.58 (0.37 to 0.77)	0.60 (0.11 to 3.39)	0.56
Any part						
Baseline	17 (54)	18 (61)	0.59 (0.45 to 0.72)	0.62 (0.49 to 0.74)	-	
3 months	15 (32)	18 (46)	0.56 (0.38 to 0.73)	0.59 (0.44 to 0.72)	0.85 (0.45 to 1.59)	0.61
6 months	16 (37)	18 (45)	0.70 (0.53 to 0.83)	0.58 (0.43 to 0.72)	0.42 (0.21 to 0.84)	**0.01**
12 months	14 (30)	18 (43)	0.63 (0.44 to 0.79)	0.51 (0.36 to 0.66)	0.55 (0.16 to 1.92)	0.35

CI=confidence interval .* Convergence not achieved for the model, Independent correlation structure used [a] Adjusted difference at follow up between treatment groups with 95% confidence interval, P value ; adjusted for cluster effect, baseline value and stratification categories (cluster size ≤4 and cluster size >4). [b] Scale for all responses ranges from 1=Yes; 0=No.[c] Scale for all responses ranges from 1=no pain to 10=most imagined pain.

Supplementary Table 3 Changes in work-related outcomes at follow up between participants randomised to usual practice (control) or to the SMArT Work intervention

	Number of offices (participants)		Mean change from baseline (95% CI)		Adjusted difference at follow-up [a]	
	Control	Intervention	Control	Intervention	Coefficient (95% CI)	P value
**Work engagement** [b]						
Vigor						
Baseline	18 (66)	19 (75)	3.28 (2.94 to 3.61)	3.37 (3.14 to 3.61)	-	
3 months	17 (47)	19 (56)	3.33 (2.95 to 3.71)	3.62 (3.32 to 3.91)	0.21 (−0.12 to 0.54)	0.21
6 months	18 (47)	19 (59)	3.10 (2.75 to 3.46)	3.64 (3.38 to 3.90)	0.44* (0.20 to 0.67)	**0.001**
12 months	16 (43)	19 (59)	3.08 (2.72 to 3.43)	3.67 (3.41 to 3.94)	0.57 (0.31 to 0.83)	**0.001**
Dedication						
Baseline	18 (66)	19 (75)	4.33 (4.03 to 4.63)	4.31 (4.08 to 4.55)	-	
3 months	17 (47)	19 (55)	4.19 (3.84 to 4.54)	4.31 (4.03 to 4.59)	0.14 (−0.19 to 0.47)	0.41
6 months	18 (47)	19 (59)	4.03 (3.71 to 4.36)	4.18 (3.92 to 4.44)	0.13* (−0.09 to 0.34)	0.24
12 months	16 (44)	19 (59)	3.87 (3.50 to 4.24)	4.33 (4.07 to 4.59)	0.42* (0.18 to 0.66)	**0.001**
Absorption						
Baseline	18 (66)	19 (75)	4.27 (3.98 to 4.55)	4.32 (4.09 to 4.54)	-	
3 months	17 (47)	19 (55)	4.28 (3.95 to 4.59)	4.47 (4.21 to 4.72)	0.08 (−0.18 to 0.34)	0.56
6 months	18 (47)	19 (59)	4.11 (3.76 to 4.45)	4.38 (4.17 to 4.60)	0.15 (−0.19 to 0.50)	0.38
12 months	16 (44)	19 (59)	4.14 (3.80 to 4.49)	4.57 (4.39 to 4.77)	0.30 (0.11 to 0.50)	**0.003**
Overall						
Baseline	18 (66)	19 (75)	3.96 (3.68 to 4.24)	4.00 (3.80 to 4.21)	-	
3 months	17 (47)	19 (56)	3.93 (3.61 to 4.26)	4.07 (3.80 to 4.35)	0.06 (−0.22 to 0.35)	0.66
6 months	18 (47)	19 (59)	3.75 (3.42 to 4.07)	4.07 (3.85 to 4.29)	0.33 (0.13 to 0.53)	**0.001**
12 months	16 (44)	19 (59)	3.67 (3.34 to 4.01)	4.19 (3.98 to 4.40)	0.44 (0.28 to 0.61)	**0.001**
**Job satisfaction** [c]						
Baseline	18 (66)	19 (77)	4.63 (4.25 to 5.02)	4.81 (4.54 to 5.07)	-	
3 months	17 (47)	19 (57)	4.79 [4.38 to 5.19)]	4.88 (4.57 to 5.18)	−0.03* (−0.23 to 0.17)	0.76
6 months	18 (48)	19 (59)	4.40 (3.98 to 4.81)	4.75 (4.46 to 5.03)	0.19* (−0.10 to 0.48)	0.20
12 months	16 (45)	19 (59)	4.62 (4.11 to 5.13)	4.63 (4.31 to 4.95)	−0.16 (−0.54 to 0.22)	0.40
**Job performance** [d]						
Baseline	18 (66)	19 (77)	5.59 (5.33 to 5.86)	5.32 (5.12 to 5.53)	-	
3 months	17 (47)	19 (57)	5.51 (5.24 to 5.78)	5.56 (5.33 to 5.80)	0.24 (−0.12 to 0.61)	0.20

6 months	18 (48)	19 (60)	5.21 (4.88 to 5.54)	5.43 (5.20 to 5.67)	0.41 (0.05 to 0.77)	**0.03**
12 months	16 (45)	19 (59)	5.31 (5.01 to 5.61)	5.51 (5.27 to 5.75)	0.53 (0.20 to 0.86)	**0.002**
**Occupational fatigue recovery** [e]						
Baseline	18 (65)	19 (76)	0.42 (0.35 to 0.48)	0.50 (0.44 to 0.56)	-	
3 months	17 (47)	19 (56)	0.38 (0.31 to 0.46)	0.40 (0.32 to 0.47)	−0.05 (−0.15 to 0.04)	0.26
6 months	18 (47)	19 (60)	0.43 (0.35 to 0.51)	0.35 (0.28 to 0.43)	−0.15 (−0.22 to −0.07)	**0.001**
12 months	16 (44)	19 (60)	0.48 (0.40 to 0.57)	0.40 (0.32 to 0.46)	−0.18 (−0.28 to −0.09)	**0.001**
**Sickness presenteeism** [f]						
Time management						
3 months	17 (42)	19 (54)	0.0 (−0.27 to 0.27)	0.13 (−0.08 to 0.34)	0.20 (−0.12 to 0.52)	0.23
6 months	18 (46)	19 (58)	−0.01 (−0.29 to 0.27)	0.24 (0.03 to 0.45)	0.26 (0.01 to 0.51)	**0.04**
12 months	16 (43)	19 (58)	−0.13 (−0.48 to 0.22)	0.23 (0.04 to 0.43)	0.44 (0.09 to 0.78)	**0.01**
Physical demands						
3 months	17 (42)	19 (56)	0.13 (−0.20 to 0.47)	0.15 (−0.11 to 0.41)	−0.09 (−0.44 to 0.26)	0.61
6 months	18 (44)	19 (59)	−0.06 (−0.33 to 0.21)	0.23 (−0.06 to 0.52)	0.18 (−0.13 to 0.49)	0.25
12 months	16 (41)	19 (59)	−0.15 (−0.57 to 0.28)	0.44 (0.17 to 0.71)	0.04 (−0.14 to 0.23)	0.66
Mental-Interpersonal demands						
3 months	17 (44)	19 (56)	−0.20 (−0.52 to 0.11)	0.12 (−0.04 to 0.27)	0.21 (−0.02 to 0.44)	0.07
6 months	18 (46)	19 (59)	0.06 (−0.15 to 0.28)	0.03 (−0.19 to 0.26)	−0.04 (−0.28 to 0.20)	0.74
12 months	16 (45)	19 (58)	−0.28 (−0.59 to 0.04)	0.16 (−0.02 to 0.34)	0.40 (0.02 to 0.77)	**0.04**
Output demands						
3 months	17 (44)	19 (56)	0.0 (−0.25 to 0.25)	−0.09 (−0.36 to 0.19)	0.03 (−0.36 to 0.42)	0.87
6 months	18 (46)	19 (58)	0.12 (−0.10 to 0.33)	−0.18 (−0.45 to 0.09)	−0.18 (−0.51 to 0.14)	0.27
12 months	16 (44)	19 (58)	0.13 (−0.20 to 0.45)	−0.07 (−0.31 to 0.17)	−0.08 (−0.42 to 0.26)	0.65
Overall work sickness presenteeism						
3 months	17 (45)	19 (56)	−0.12 (−0.32 to 0.08)	0.07 (−0.09 to 0.24)	0.25 (0.01 to 0.49)	**0.04**
6 months	18 (47)	19 (59)	0.0 (−0.22 to 0.22)	0.08 (−0.10 to 0.26)	0.10 (−0.15 to 0.36)	0.42
12 months	16 (45)	19 (59)	−0.12 (−0.39 to 0.15)	0.13 (−0.05 to 0.30)	0.25 (−0.08 to 0.58)	0.14
**Absenteeism self-report data**						
3 months	17 (41)	19 (52)	−0.02 (−0.06 to 0.02)	0.01 (−0.03 to 0.05)	0.02 (−0.01 to 0.05)	0.18
6 months	17 (43)	19 (54)	0.01 (−0.04 to 0.06)	−0.001 (−0.03 to 0.03)	−0.02 (−0.05 to 0.02)	0.28
12 months	16 (39)	19 (54)	0.02 (−0.07 to 0.10)	0.01 (−0.03 to 0.05)	−0.02 (−0.10 to 0.06)	0.68
Absenteeism due to other reasons						
3 months	17 (39)	19 (51)	0.07 (−0.07 to 0.22)	0.03 (−0.04 to 0.11)	−0.10 (−0.24 to 0.04)	0.16
6 months	17 (41)	19 (54)	0.03 (−0.10 to 0.15)	−0.01 (−0.09 to 0.07)	−0.05* (−0.14 to 0.04)	0.25

12 months	16 (37)	17 (52)	−0.03 (−0.11 to 0.06)	0.04 (−0.01 to 0.09)	0.04 (−0.04 to 0.13)	0.35

IQR=interquartile range; CI=confidence interval.[a] Adjusted difference at follow up between treatment groups with 95% confidence interval, P value ; adjusted for cluster effect, baseline value and stratification categories (cluster size ≤4 and cluster size >4). [b] Scale for all responses from 0=never to 6=always. A higher score indicates better engagement [c] Scale for all responses ranges from 1=dissatisfied to 7=extremely satisfied. A higher score indicate higher job satisfaction [d] Scale for all responses ranges from 1=very poorly to 7=extremely well. A higher score indicates better job performance [e] Scale for all responses yes=1 and no=0 ; A lower score indicates less occupational fatigue
[f] Scale for all responses ranges from 1=all of the time to 5=none of the time). A higher score indicates less sickness presenteeism * Convergence not achieved for the model, Independent correlation structure used.

Supplementary Table 4 Organisational sickness absenteeism records at pre-intervention (past 12 months) and at 12 months (12 months of intervention) for participants randomised to usual practice (control) or to the SMArT Work intervention

	Number of offices (participants)		Mean (95% CI)		Adjusted difference at follow-up [a]	
	**Control**	**Intervention**	**Control**	**Intervention**	**Coefficient (95% CI)**	**P value**
**Absenteeism clinical records**						
Mean No of days missed						
Pre-intervention	15 (48)	18 (50)	4.74 (−0.47 to 9.97)	4.60 (1.01 to 8.19)		
During intervention	15 (48)	18 (50)	8.31 (1.26 to 15.37)	9.02 (0.97 to 17.07)		
Mean change at 12 months	15 (48)	18 (50)	3.56 (−5.23 to 12.35)	4.42 (−4.24 to 13.08)	1.32 (−9.99 to 12.63)	0.82
Mean No of episodes						
Pre-intervention	15 (48)	18 (50)	0.88 (0.59 to 1.16)	0.56 (0.37 to 0.75)		
During intervention	15 (48)	18 (50)	0.98 (0.64 to 1.32)	1.00 (0.55 to 1.45)		
Mean change at 12 months	15 (48)	18 (50)	0.10 (−0.24 to 0.45)	0.44 (0.05 to 0.83)	0.24 (−0.35 to 0.82)	0.43

CI=confidence interval .[a] Adjusted difference at follow up between treatment groups with 95% confidence interval, P value ; adjusted for cluster effect, baseline value and stratification categories (cluster size≤4 & cluster size>4).

Supplementary Table 5 Changes in cognitive function variables at 3, 6 and 12 months follow up between participants randomised to usual practice (control) or to the SMArT Work intervention

	Number of offices (participants)		Mean change from baseline (95% CI)		Adjusted difference at follow-up [a]	
	**Control**	**Intervention**	**Control**	**Intervention**	**Coefficient (95% CI)**	**P-value**
**Verbal learning (memory)[b]**						
3 months	17 (50)	19 (66)	0.26 (−0.94 to 1.46)	1.27 (0.14 to 2.41)	−0.12 (−1.95 to 1.71)	0.90
6 months	17 (45)	19 (58)	1.96 (0.85 to 3.06)	2.50 (1.48 to 3.52)	0.62 (−0.86 to 2.11)	0.41
12 months	15 (42)	19 (62)	3.97 (2.85 to 5.10)	4.87 (3.89 to 5.84)	0.35 (−1.07 to 1.77)	0.63
**Verbal fluency[b]**						
3 months	17 (50)	19 (66)	1.52 (0.49 to 2.54)	1.15 (0.32 to 1.98)	−0.57 (−1.86 to 0.72)	0.39
6 months	17 (45)	19 (58)	2.38 (1.46 to 3.29)	1.36 (0.31 to 2.41)	−1.12 (−2.43 to 0.19)	0.09
12 months	15 (43)	19 (62)	1.72 (0.66 to 2.78)	2.05 (1.11 to 2.98)	0.25 (−0.92 to 1.41)	0.68
**DSST[b]**						
Level1						
3 months	18 (50)	19 (65)	4.44 (3.06 to 5.81)	3.62 (2.38 to 4.84)	−0.04 (−1.31 to 1.22)	0.95
6 months	17 (46)	19 (57)	6.06 (4.63 to 7.50)	5.65 (4.36 to 6.94)	−0.37 (−1.94 to 1.19)	0.65
12 months	16 (44)	19 (61)	5.55 (4.05 to 7.04)	5.39 (3.75 to 7.04)	−0.84 (−2.58 to 0.90)	0.35
Level2						
3 months	18 (50)	19 (66)	2.62 (1.14 to 4.09)	2.58 (1.49 to 3.66)	−0.14 (−2.11 to 1.84)	0.89
6 months	17 (46)	19 (58)	3.41 (1.87 to 4.95)	4.19 (3.23 to 5.15)	0.65 (−0.91 to 2.21)	0.41
12 months	16 (44)	19 (62)	2.29 (0.55 to 3.93)	4.05 (2.83 to 5.27)	1.44 (−0.38 to 3.26)	0.12
**Stroop[c]**						
Reaction time (Congruent Level)						
3 months	18 (50)	19 (65)	−54.92 (−98.9 to −10.89)	−132.7 (−172.9 to −92.4)	−70.9 (−119.1 to −22.8)	**0.004**
6 months	17 (46)	19 (57)	−98.8 (−140.6 to −56.9)	−170.8 (−213.2 to −128.3)	−65.2 (−118.2 to −12.2)	**0.02**
12 months	16 (44)	19 (61)	−107.5 (−146.3 to −68.8)	−154.8 (−196.7 to −112.9)	−34.4 (−65.9 to −2.79)	**0.03**
Reaction time (Incongruent level)						
3 months	18 (50)	19 (66)	−111.9 (−173.1 to −50.8)	−107.6 (−153.8 to −61.5)	4.88 (−69.16 to 78.90)	0.90

6 months	17 (46)	19 (58)	−193.1(−246.5 to −139.7)	−217.8(−264.1 to −171.7)	−27.63 (−95.52 to 40.25)	0.43
12 months	16 (44)	19 (62)	−245.1(−301.8 to −188.4)	−248.3(−293.5 to −203.2)	−2.13 (−66.40 to 62.14)	0.95
Proportion correct hits (Congruent Level)[b]						
3 months	18 (50)	19 (65)	0.01 (−0.003 to 0.02)	−0.001 (−0.01 to 0.01)	−0.003 (−0.01 to 0.004)	0.33
6 months	17 (46)	19 (57)	0.002 (−0.01 to 0.014)	0.004 (−0.01 to 0.01)	0.003 (−0.001 to 0.01)	0.11
12 months	16 (44)	19 (61)	−0.003 (−0.01 to 0.008)	0.003 (−0.004 to 0.01)	0.007 (−0.003 to 0.02)	0.20
Proportion correct hits (Incongruent Level)[b]						
3 months	18 (50)	19 (66)	0.012 (−0.007 to 0.03)	0.03 (−0.003 to 0.06)	−0.02 (−0.03 to −0.002)	**0.02**
6 months	17 (46)	19 (58)	0.002 (−0.02 to 0.02)	0.04 (0.003 to 0.07)	−0.001 (−0.014 to 0.012)	0.87
12 months	16 (44)	19 (62)	0.015 (−0.004 to 0.04)	0.03 (−0.004 to 0.06)	−0.01 (−0.03 to 0.004)	0.14

CI=confidence interval. DSST=Digital Simple Substitution. Congruent = ink colour and word refer to the same colour. Incongruent = ink colour and word don't match. [a] Adjusted for cluster effect, baseline values, stratification category (cluster size≤4 & cluster size>4). [b] A higher number indicates better performance. [c] A lower number indicates a better performance.

Supplementary Table 6 Changes in mood states at follow up times at work place for participants randomised to usual practice (control) or to the SMArT Work intervention

	Number of Offices (participants)		Mean score change from baseline 95% CI)		Adjusted difference at follow-up[a]	
	**Control**	**Intervention**	**Control**	**Intervention**	**Coefficient (95% CI)**	**P-value**
Anxiety today[b]						
3 months	17 (45)	19 (56)	0.00 (−0.04 to 0.04)	−0.02 (−0.06 to 0.02)	−0.02 (−0.06 to 0.03)	0.51
6 months	18 (47)	19 (58)	0.02 (−0.02 to 0.07)	−0.03 (−0.07 to 0.00)	−0.06* (−0.09 to −0.02)	**0.002**
12 months	18 (50)	19 (64)	0.03 (−0.02 to 0.07)	−0.02 (−0.06 to 0.01)	−0.05 (−0.10 to −0.00)	**0.04**
Anxiety generally[b]						
3 months	17 (45)	19 (56)	−0.04 (−0.09 to −0.00)	−0.01 (−0.04 to 0.02)	0.05 (0.01 to 0.08)	**0.02**
6 months	18 (47)	19 (58)	−0.02 (−0.07 to 0.04)	−0.05 (−0.10 to 0.01)	−0.01 (−0.06 to 0.04)	0.66
12 months	16 (45)	19 (59)	−0.02 (−0.06 to 0.02)	−0.03 (−0.08 to 0.02)	0.02 (−0.03 to 0.07)	0.39
Depression today[b]						
3 months	17 (45)	19 (56)	0.01 (−0.01 to 0.03)	−0.02 (−0.05 to 0.00)	−0.03 (−0.06 to 0.01)	0.14

6 months	18 (47)	19 (58)	0.00 (−0.02 to 0.02)	−0.02 (−0.04 to −0.01)	−0.03 (−0.05 to 0.00)	0.05
12 months	16 (45)	19 (59)	−0.00 (−0.03 to 0.03)	0.00 (−0.03 to 0.03)	0.00 (−0.03 to 0.03)	0.91
Depression generally[b]						
3 months	17 (45)	19 (56)	−0.01 (−0.03 to 0.00)	−0.01 (−0.03 to 0.02)	0.02 (−0.01 to 0.04)	0.24
6 months	18 (47)	19 (58)	−0.00 (−0.04 to 0.03)	−0.01 (−0.04 to 0.02)	0.00 (−0.04 to 0.04)	0.94
12 months	16 (45)	19 (59)	−0.02 (−0.05 to 0.02)	−0.00 (−0.03 to 0.02)	0.02 (−0.01 to 0.05)	0.18
Hostility today[b]						
3 months	17 (45)	19 (56)	−0.03 (−0.09 to 0.03)	−0.02 (−0.7 to 0.03)	−0.01 (−0.07 to 0.04)	0.64
6 months	18 (47)	19 (58)	−0.01 (−0.07 to 0.04)	0.00 (−0.05 to 0.05)	−0.00 (−0.05 to 0.05)	0.93
12 months	16 (45)	19 (59)	−0.04 (−0.10 to 0.02)	0.01 (−0.04 to 0.05)	0.04 (−0.02 to 0.09)	0.18
Hostility generally[b]						
3 months	17 (45)	19 (56)	0.00 (−0.05 to 0.05)	0.03 (−0.01 to 0.06)	0.03 (−0.01 to 0.07)	0.17
6 months	18 (47)	19 (58)	−0.02 (−0.06 to 0.02)	0.01 (−0.03 to 0.05)	0.03 (−0.01 to 0.08)	0.13
12 months	16 (45)	19 (59)	−0.04 (−0.09 to 0.02)	−0.00 (−0.04 to 0.03)	0.06 (0.02 to 0.10)	**0.008**
Dysphoria today[b]						
3 months	17 (45)	19 (56)	−0.01 (−0.03 to 0.02)	−0.02 (−0.05 to 0.01)	−0.02 (−0.05 to 0.02)	0.35
6 months	18 (47)	19 (58)	0.01 (−0.02 to 0.04)	−0.02 (−0.04 to 0.01)	−0.03* (−0.05 to −0.00)	**0.03**
12 months	18 (50)	19 (64)	−0.01 (−0.04 to 0.02)	−0.01 (−0.03 to 0.02)	−0.00 (−0.04 to 0.04)	0.94
Dysphoria generally[b]						
3 months	17 (45)	19 (56)	−0.02 (−0.05 to 0.01)	0.00 (−0.02 to 0.03)	0.04 (0.01 to 0.06)	**0.001**
6 months	18 (47)	19 (58)	−0.02 (−0.05 to 0.02)	−0.02 (−0.05 to 0.02)	0.01 (−0.04 to 0.05)	0.77
12 months	16 (45)	19 (59)	−0.03 (−0.06 to 0.01)	−0.01 (−0.04 to 0.01)	0.02 (−0.01 to 0.06)	0.16
Positive affect today[b]						
3 months	17 (45)	19 (56)	0.01 (−0.07 to 0.09)	0.03 (−0.04 to 0.10)	0.03 (−0.06 to 0.12)	0.54
6 months	18 (47)	19 (59)	0.03 (−0.03 to 0.09)	0.04 (−0.04 to 0.12)	0.00 (−0.09 to 0.09)	0.97
12 months	16 (45)	19 (59)	0.05 (−0.03 to 0.13)	0.06 (−0.02 to 0.14)	−0.01 (−0.09 to 0.07)	0.77
Positive affect generally[b]						
3 months	17 (45)	19 (56)	0.03 (−0.03 to 0.10)	0.09 (0.03 to 0.16)	0.05* (−0.01 to 0.11)	0.13
6 months	18 (47)	19 (59)	−0.03 (−0.09 to 0.03)	0.05 (−0.02 to 0.11)	0.07 (−0.02 to 0.15)	0.11
12 months	16 (45)	19 (59)	0.03 (−0.04 to 0.11)	0.05 (−0.02 to 0.12)	−0.01 (−0.10 to 0.08)	0.82
Sensation seeking today[b]						
3 months	17 (45)	19 (56)	0.02 (−0.01 to 0.05)	0.03 (−0.02 to 0.07)	0.00 (−0.04 to 0.05)	0.84
6 months	18 (47)	19 (59)	−0.00 (0.03 to 0.03)	0.01 (−0.03 to 0.06)	0.02 (−0.04 to 0.07)	0.52
12 months	16 (45)	19 (59)	0.03 (−0.01 to 0.06)	0.02 (−0.02 to 0.06)	−0.02 (−0.05 to 0.02)	0.32
Sensation seeking generally[b]						
3 months	17 (45)	19 (56)	0.03 (−0.01 to 0.06)	0.07 (0.02 to 0.11)	0.03* (−0.01 to 0.07)	0.18
6 months	18 (47)	19 (59)	0.01 (−0.03 to 0.04)	0.04 (0.00 to 0.08)	0.03 (−0.02 to 0.08)	0.28
12 months	16 (45)	19 (59)	−0.01 (−0.06 to 0.03)	0.03 (−0.01 to 0.07)	0.03 (−0.02 to 0.07)	0.26

PASS today [b]						
3 months	17 (45)	19 (56)	0.01 (−0.04 to 0.06)	0.03 (−0.02 to 0.08)	0.02 (−0.04 to 0.08)	0.54
6 months	18 (47)	19 (59)	0.01 (−0.02 to 0.05)	0.03 (−0.03 to 0.08)	0.01 (−0.06 to 0.08)	0.74
12 months	16 (45)	19 (59)	0.04 (−0.01 to 0.09)	0.04 (−0.01 to 0.09)	−0.03 (−0.06 to 0.01)	0.13
PASS generally [b]						
3 months	17 (45)	19 (56)	0.03 (−0.01 to 0.07)	0.08 (0.03 to 0.13)	0.04 (−0.00 to 0.08)	0.07
6 months	18 (47)	19 (59)	−0.01 (−0.05 to 0.03)	−0.04 (−0.00 to 0.09)	0.05 (−0.01 to 0.11)	0.10
12 months	16 (45)	19 (59)	0.01 (−0.04 to 0.06)	0.04 (−0.01 to 0.08)	0.00 (−0.05 to 0.05)	0.97

CI=confidence interval; PASS=Positive Affect+Sensation Seeking; Dysphoria=Anxiety+Depression+Hostility. [a] Adjusted difference at follow up between treatment groups with 95% confidence interval, P value ; adjusted for cluster effect, baseline value and stratification categories (cluster size ≤4 and cluster size >4). [b] 1 point is given for each item that is ticked within the mood state. The more items ticked the higher the score on that mood state

Supplementary Table 7 Changes in quality of life at follow up for participants randomised to usual practice (control) or to the SMArT Work intervention

	**Number of Offices (participants)**		**Mean score change from baseline (95% CI)**		**Adjusted difference at follow-up** [a]	
	**Control**	**Intervention**	**Control**	**Intervention**	**Coefficient (95% CI)**	**P-value**
**Quality of life** [b]**:**						
Physical health						
Baseline	18 (66)	19 (77)	-	-	-	
3 months	17 (46)	19 (57)	0.08 (−0.04 to 0.20)	0.21 (0.09 to 0.32)	0.10 (−0.05 to 0.26)	0.20
6 months	18 (47)	19 (60)	0.04 (−0.08 to 0.16)	0.18 (0.04 to 0.31)	0.13 (−0.06 to 0.31)	0.17
12 months	16 (45)	19 (60)	0.03 (−0.10 to 0.15)	0.18 (0.05 to 0.31)	0.14 (−0.04 to 0.31)	0.12
Psychological †						
Baseline	18 (66)	19 (77)	-	-	-	
3 months	17 (46)	19 (57)	0.06 (−0.07 to 0.19)	0.21 (0.11 to 0.32)	0.14 (−0.04 to 0.31)	0.13
6 months	18 (47)	19 (60)	−0.07 (−0.22 to 0.07)	0.18 (0.06 to 0.31)	0.22 (0.04 to 0.41)	**0.02**
12 months	16 (45)	19 (60)	−0.10 (−0.24 to 0.05)	0.15 (0.01 to 0.28)	0.18 (0.03 to 0.33)	**0.02**
Social relationship						
Baseline	18 (66)	19 (77)	-	-	-	
3 months	17 (46)	19 (57)	−0.01 (−0.22 to 0.19)	0.06 (−0.11 to 0.23)	0.04 (−0.22 to 0.29)	0.79
6 months	18 (47)	19 (60)	−0.04 (−0.22 to 0.13)	0.05 (−0.12 to 0.22)	0.09 (−0.12 to 0.31)	0.41
12 months	16 (45)	19 (60)	0.00 (−0.22 to 0.22)	0.07 (−0.08 to 0.23)	0.06 (−0.19 to 0.30)	0.64
Environment						
Baseline	18 (66)	19 (77)	-	-	-	
3 months	17 (46)	19 (57)	0.11 (0.01 to 0.21)	0.14 (0.02 to 0.27)	−0.01 (−0.13 to 0.11)	0.85
6 months	18 (47)	19 (60)	−0.002(−0.14 to 0.14)	0.18 (0.08 to 0.29)	0.16 (0.01 to 0.32)	**0.04**

12 months	16 (45)	19 (60)	0.06 (−0.08 to 0.20)	0.14 (0.03 to 0.25)	0.08 (0.03 to 0.13)	**0.003**
Overall Quality of life						
Baseline	18 (66)	19 (77)	-	-	-	
3 months	17 (46)	19 (57)	0.07 (−0.01 to 0.15)	0.17 (0.08 to 0.25)	0.07 (−0.03 to 0.18)	0.17
6 months	18 (47)	19 (60)	−0.01 (−0.10 to 0.08)	0.16 (0.07 to 0.26)	0.16 (0.06 to 0.27)	**0.002**
12 months	16 (45)	19 (60)	0.01 (−0.09 to 0.10)	0.14 (0.05 to 0.24)	0.12 (0.00 to 0.23)	**0.05**

CI=confidence interval. [a] Adjusted difference at follow up between treatment groups with 95% confidence interval, P value; adjusted for cluster effect, baseline value and stratification categories (cluster size ≤4 and cluster size >4). [b] Scale for all responses ranges from 1=very dissatisfied to 5=very satisfied. A higher score indicates a better quality of life † Q26 is not included in this domain due to the duplication of Q25.
